# Omics- and Pharmacogenomic Evidence for the Prognostic, Regulatory, and Immune-Related Roles of PBK in a Pan-Cancer Cohort

**DOI:** 10.3389/fmolb.2021.785370

**Published:** 2021-11-11

**Authors:** Yi Liu, Juan Xiang, Gang Peng, Chenfu Shen

**Affiliations:** ^1^ Department of Neurosurgery, Xiangya Hospital, Central South University, Changsha, China; ^2^ Department of Geriatrics, Xiangya Hospital, Central South University, Changsha, China

**Keywords:** PBK, immune infiltration, methylation, cell cycle, tumorigenesis, pan-cancer

## Abstract

PDZ-binding kinase (PBK) is known to regulate tumor progression in some cancer types. However, its relationship to immune cell infiltration and prognosis in different cancers is unclear. This was investigated in the present study by analyzing data from TCGA, GEO, GETx, TIMER, CPTAC, GEPIA2, cBioPortal, GSCALite, PROGNOSCAN, PharmacoDB, STRING, and ENCORI databases. PBK was overexpressed in most tumors including adenocortical carcinoma (hazard ratio [HR] = 2.178, *p* < 0.001), kidney renal clear cell carcinoma (KIRC; HR = 1.907, *p* < 0.001), kidney renal papillary cell carcinoma (HR = 3.024, *p* < 0.001), and lung adenocarcinoma (HR = 1.255, *p* < 0.001), in which it was associated with poor overall survival and advanced pathologic stage. PBK methylation level was a prognostic marker in thyroid carcinoma (THCA). PBK expression was positively correlated with the levels of BIRC5, CCNB1, CDC20, CDK1, DLGAP5, MAD2L1, MELK, PLK1, TOP2A, and TTK in 32 tumor types; and with the levels of the transcription factors E2F1 and MYC, which regulate apoptosis, the cell cycle, cell proliferation and invasion, tumorigenesis, and metastasis. It was also negatively regulated by the microRNAs hsa-miR-101-5p, hsa-miR-145-5p, and hsa-miR-5694. PBK expression in KIRC, liver hepatocellular carcinoma, THCA, and thymoma was positively correlated with the infiltration of immune cells including B cells, CD4+T cells, CD8^+^ T cells, macrophages, monocytes, and neutrophils. The results of the functional enrichment analysis suggested that PBK and related genes contribute to tumor development via cell cycle regulation. We also identified 20 drugs that potentially inhibit PBK expression. Thus, PBK is associated with survival outcome in a variety of cancers and may promote tumor development and progression by increasing immune cell infiltration into the tumor microenvironment. These findings indicate that PBK is a potential therapeutic target and has prognostic value in cancer treatment.

## Introduction

PDZ-binding kinase (PBK), also known as T lymphokine-activated killer cell-derived protein kinase (TOPK), is a serine-threonine kinase of the mitogen-activated protein kinase (MAPKK) family that regulates cell survival, proliferation, growth, apoptosis, and inflammation (Abe et al., 2000; Gaudet et al., 2000; Zhao et al., 2020). As a mitotic kinase, PBK is activated by the cyclin-dependent kinase 1 (CDK1)/cyclin B1 complex during mitosis and promotes cytokinesis by phosphorylating polycomb inhibitory complex 1 (PRC1) or anaphase-promoting complex (APC) ubiquitin ligase (Kraft et al., 2003; Abe et al., 2007). *PBK* knockdown was shown to result in cytokinesis defects in cancer cells (Park et al., 2010).

PBK is overexpressed in a variety of active proliferating cells including malignant tumor cells as well as normal sperm cells. The upregulation of PBK has diagnostic and prognostic significance in cancer. For example, increased expression of PBK has been linked to poor prognosis in patients with ovarian serous cystadenocarcinoma (OV), esophageal carcinoma (ESCA), and stomach adenocarcinoma (STAD) (Ikeda et al., 2016; Ohashi et al., 2016; Ohashi et al., 2017). PBK is also related to cell transformation induced by H-Ras, activation of c-Jun-NH2 kinase (JNK), and expression of p53 induced by ultraviolet A, ultraviolet B, and DNA damage, respectively (Oh et al., 2007; Hu et al., 2010). PBK directly phosphorylates extracellular signal-regulated kinase (ERK), histone H3 (Ser10), histone H2AX (Ser139), peroxide-reduced protein 1 (PRX1, Ser32), JNK1 (Thr183/Tyr185), and p53-associated protein kinase (PRPK, Ser250) (Zykova et al., 2010; Roh et al., 2018) and activates downstream signaling pathways including MAPK, ribosomal S6 kinase (RSK), and transcription factors (TFs) such as activating protein 1 (AP-1) or nuclear factor kappa B (NF-κB) through phosphorylation, thereby promoting cell proliferation, migration, and invasion (Aksamitiene et al., 2010; Park et al., 2014; Ishikawa et al., 2018). Additionally, PBK promotes cell migration by regulating phosphatidylinositol 3-kinase (PI3K)/phosphatase and tensin homolog (PTEN)/protein kinase B (AKT) or transforming growth factor beta 1 (TGF-β1)/mothers against decapentaplegic (Smad) signaling pathways (Shih et al., 2012; Lee et al., 2020). PBK is differentially expressed between cancer and normal tissues and modulates radiosensitivity in tumors, suggesting that patients can be treated with radiotherapy (Pirovano et al., 2017); and a recent retrospective analysis showed that PBK expression was negatively correlated with the prognosis of patients with oral squamous cell carcinoma treated with radiotherapy (Yu et al., 2021). The PBK inhibitor OTS964/OTS514 was shown to inhibit cytokinesis and cancer cell growth (Matsuo et al., 2014), and OTS964/OTS514 induced the apoptosis of lung cancer cells including small cell lung cancer (SCLC) cells (Park et al., 2017). Thus, PBK likely plays an important role in cancer development.

Previous studies on PBK were limited to a single cancer type and lacked a comparative analysis of multiple tumors. The aim of the present study was to examine the role of PBK in different cancers and elucidate the molecular mechanisms and functions of PBK and its interacting molecules in carcinogenesis. We also analyzed the relationships between PBK expression, mutation and methylation status, clinical outcome, and tumor immune infiltration in cancers, and screened drugs that can potentially inhibit PBK. Our results demonstrate that PBK plays an important role in carcinogenesis and is a potential therapeutic target in cancer treatment.

## Materials and Methods

### Gene Expression Analysis

RNA expression and clinical data in The Cancer Genome Atlas (TCGA) and Genotype-Tissue Expression (GTEx) databases were downloaded from the University of California at Santa Cruz (UCSC) Xena database (https://xenabrowser.net/datapages/). The R “limma” package was used to analyze the mRNA expression of PBK in the pan-cancer cohort.

The UALCAN portal (http://ualcan.path.uab.edu/analysis-prot.html) was used to access the Clinical Proteome Oncology Analysis Alliance (CPTAC) dataset (Chen F. et al., 2019), which includes breast invasive carcinoma (BRCA), colon adenocarcinoma (COAD), OV, clear cell renal cell carcinoma, endometrial carcinoma (UCEC), lung adenocarcinoma (LUAD), and childhood brain cancer. We used these data to compare total protein expression between primary tumors and normal tissue by entering “PBK” into the search field.

We used the Gene Expression Profiling Interactive Analysis v2 (GEPIA2; http://gepia2.cancer-pku.cn/#index) “Pathological Stage Plot” module to obtain maps of PBK expression in different pathologic stages (stage I–IV) of TCGA tumors. Log2 (transcripts per million+1) expression data and an adjusted *p* value < 0.05 were used as cutoffs to generate the stage diagram.

### Alterations in Gene Expression

We investigated the inheritance of *PBK* gene modifications in different cancers using the cBioPortal portal (https://www.cbioportal.org/) (Gao et al., 2013) through “TCGA Pan-Cancer Atlas Studies” in the “Quick select” section with “PBK” as query. The results of genetic changes in TCGA tumor samples were displayed in the “Cancer Types Summary” module, including the type of mutation and copy number alterations. The comparison module also returned disease specificity and overall survival (OS), progression-free survival (PFS), and disease-free survival (DFS) in TCGA cancer samples with or without *PBK* gene mutations. A log-rank *p* value < 0.05 was used as the cutoff.

### PBK Regulatory Networks of TFs, microRNAs (miRNAs), and Methylation

In order to identify regulatory factors that affect PBK expression, we used Harmonizome (https://maayanlab.cloud/Harmonizome) (Rouillard et al., 2016) to predict TFs that can bind to the *PBK* gene promoter in the Chromatin Immunoprecipitation Enrichment Analysis (ChEA), Encyclopedia of DNA Elements (ENCODE), JASPAR, and TRANScription FACtor (TRANSFAC) databases. TFs that appeared at least twice in these databases were re-screened in the ENCORI database (http://starbase.sysu.edu.cn/panCancer.php) (*p* < 0.05, R = 1 to −1).

miRNAs targeting PBK were identified using the microRNA database (miRDB) (http://mirdb.org/cgi-bin/search.cgi) and TargetScan (http://www.targetscan.org/vert_72/). miRNAs that appeared at least twice were re-screened in ENCORI to determine their relationship to PBK (*p* < 0.05, R = 1 to −1).

Gene Set Cancer Analysis (GSCALite; http://bioinfo.life.hust.edu.cn/web/GSCALite/) is a bioinformatics platform that allows methylation, drug sensitivity, genetic, gene variation, and survival analyses of cancer datasets (Liu et al., 2018). We used the GSCALite platform to analyze the differential methylation of PBK and TFs between tumors and paired normal tissues in different types of cancer in TCGA. After logging onto the GSCALite server, we used the “TCGA Cancer–methylation” module to select 32 TCGA cancer types. In the final map generated by GSCALite, only 14 cancer types showed methylation profiles of PBK and related TFs. Using the same platform, we analyzed the relationship between methylation and the expression of PBK and related TFs in different cancers.

### Survival Analysis

The PrognoScan database (http://dna00.bio.kyutech.ac.jp/PrognoScan/index.html) allows meta-analysis of the prognostic value of genes by comparing the relationship between gene expression and variables of interest in a large number of published reports (Mizuno et al., 2009). We used this platform to evaluate the relationship between PBK expression and patient prognosis in different cancer cohorts. Survival curves were generated with PrognoScan and the “survival” package in R from TCGA data. The survival results are shown as hazard ratio (HR) with 95% confidence interval (CI) and log-rank *p* value.

### Correlation Analysis

Pearson correlation analysis was performed to evaluate the correlation between PBK and immune checkpoints, tumor mutation burden (TMB), microsatellite instability (MSI), DNA methylation, and mismatch repair (MMR). The “heatmap” package in R was used to display the results as a heatmap.

### Immune Cell Infiltration Into the Tumor Microenvironment

The Tumor IMmune Estimation Resource (TIMER; http://timer.cistrome.org/) (Li et al., 2017) web server is a comprehensive resource for systematic analysis of immune cell infiltration in different cancer types. We used the immune gene module of TIMER to assess the correlation between PBK expression in six immune cell types and their infiltration in 32 TCGA tumors. We also used the Tumor-Immune System Interactions database (TISIDB) (http://cis.hku.hk/TISIDB/) to evaluate whether there was a significant difference in PBK expression between responders and non-responders to immunotherapies (e.g., anti-programmed death 1 [PD1] or anti-programmed death ligand 1 [PD-L1]).

### Enrichment Analysis of PBK-Related Genes

We used the Search Tool for the Retrieval of Interacting Genes/Proteins (STRING) website (https://string-db.org/) with “PBK” and organism = *Homo sapiens* to construct a network of the interaction between PBK and related genes. We then used the GSCALite platform to analyze the function of PBK-related genes by Gene Ontology (GO) and Kyoto Encyclopedia of Genes and Genomes (KEGG) pathway enrichment analyses. Using the ggplot2 package in R v3.6.2, a point map was generated to visualize the results (*p* < 0.05).

### Drugs Targeting PBK

We downloaded data on PBK-related drugs from PharmacoDB (https://www.pharmacodb.pmgenomics.ca/) (Smirnov et al., 2018), with *p* < 0.01 and a standardized coefficient of −1 to 1 as thresholds. The “heatmap” package in R was used to display the results as a heatmap.

## Results

### Expression of PBK in Human Cancers

**FIGURE 1:**
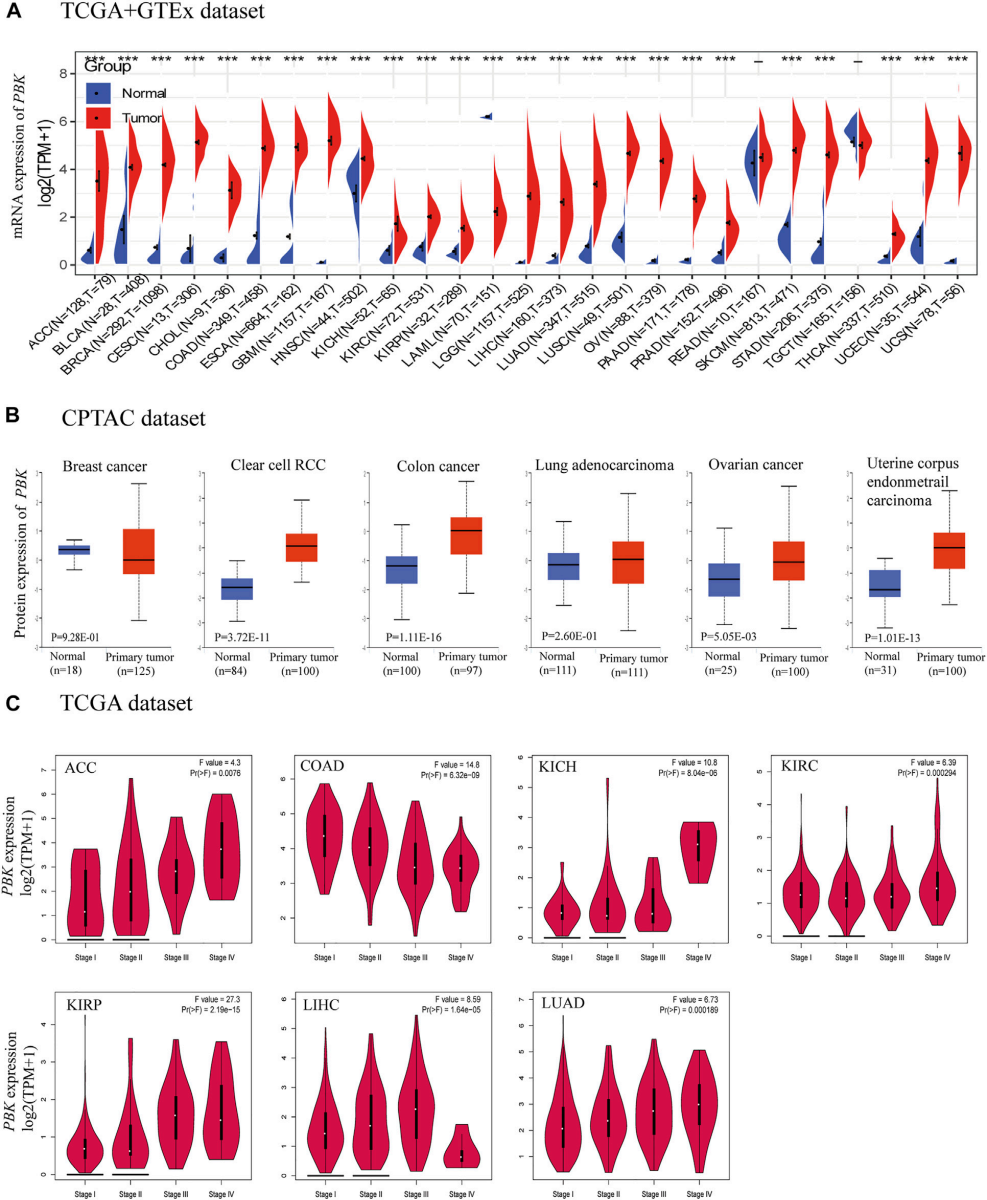
PBK expression. **(A)** Expression of PBK in tumor and normal tissues in TCGA + GETx dataset. **(B)** PBK protein level in tumor tissues compared to normal tissues. **(C)** PBK expression in different stages of ACC, COAD, KICH, KIRC, KIRP, LIHC, and LUAD.

We analyzed the differential expression of PBK in 33 cancers as compared to normal tissue using TCGA and GTEx databases. PBK expression was higher in adrenocortical carcinoma (ACC), bladder urothelial carcinoma (BLCA), breast invasive carcinoma (BRCA), cervical squamous cell carcinoma and endocervical adenocarcinoma (CESC), cholangiocarcinoma (CHOL), colon adenocarcinoma (COAD), esophageal carcinoma (ESCA), glioblastoma multiforme (GBM), head and neck squamous cell carcinoma (HNSC), kidney chromophobe (KICH), kidney renal clear cell carcinoma (KIRC), kidney renal papillary cell carcinoma (KIRP), brain lower grade glioma (LGG), liver hepatocellular carcinoma (LIHC), lung adenocarcinoma (LUAD), lung squamous cell carcinoma (LUSC), ovarian serous cystadenocarcinoma (OV), rectum adenocarcinoma (READ), prostate adenocarcinoma (PRAD), skin cutaneous melanoma (SKCM), stomach adenocarcinoma (STAD), thyroid carcinoma (THCA), uterine corpus endometrial carcinoma (UCEC), and uterine carcinosarcoma (UCS) but lower in acute myeloid leukemia (LAML) compared to normal tissues (all *p* < 0.01; Figure 1A).

Analysis of the CPTAC dataset showed that PBK protein expression was significantly higher in KIRC, COAD, OV, and UCEC tissues than in normal tissues (Figure 1B). The results of the pathologic staging map based on GEPIA2 data revealed significant associations between PBK expression and pathologic stage in ACC (*p* = 0.0076), COAD (*p* = 6.32e−09), KICH (*p* = 8.04e−06), KIRC (*p* = 0.000294), KIRP (*p* = 2.19e−15), LIHC (*p* = 1.64e−05), and LUAD (*p* = 0.000189) (Figure 1C). In ACC, KICH, KIRC, KIRP, and LUAD, the association was positive whereas in COAD and LIHC, there was an inverse relationship between pathologic stage and PBK expression.

### *PBK* Mutation Profile in Different Tissues

**FIGURE 2:**
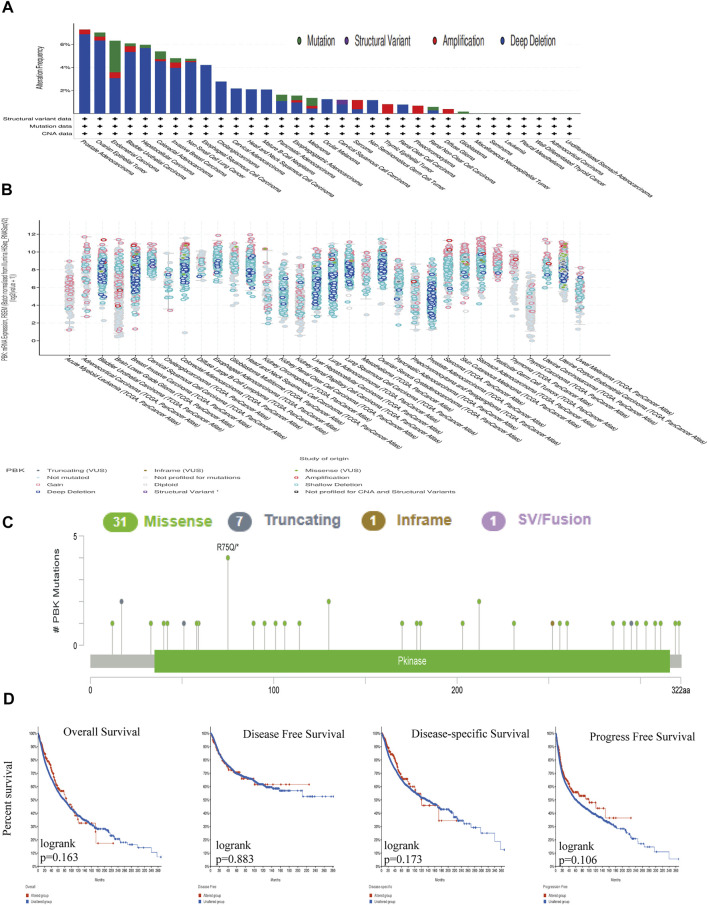
Genetic alterations in PBK. **(A)** PBK mutation frequency in multiple TCGA pan-cancer dataset in the cBioPortal database. **(B)** Overall mutation count for CD96 in various TCGA cancer types in the cBioPortal database. **(C)** Mutation diagram of CD96 across protein domains in different cancer types. **(D)** OS, DSS, DFS, and PFS in cancers with genetic alterations in PBK.

We used cBioPortal to determine the mutation frequency of *PBK* in the TCGA database (10,967 samples in 32 studies). We found that PBK had a relatively high mutation frequency in PRAD, OV, and UCEC (>6%) (Figures 2A,B). A total of 40 mutations (including 31 missense, 7 truncation, 1 deletion, and 1 fusion) were detected between amino acid 0 and 322 (Figure 2C).

*PBK* gene mutations were unrelated to survival and prognosis of cancers in TCGA. Using cBioPortal, we determined that there was no significant difference in OS (*p* = 0.163), DFS (*p* = 0.883), disease-specific survival (DSS; *p* = 0.173), or PFS (*p* = 0.106) between samples with vs those without *PBK* mutation (Figure 2D).

### Association Between *PBK* Expression and Cancer Patient Prognosis

**FIGURE 3:**
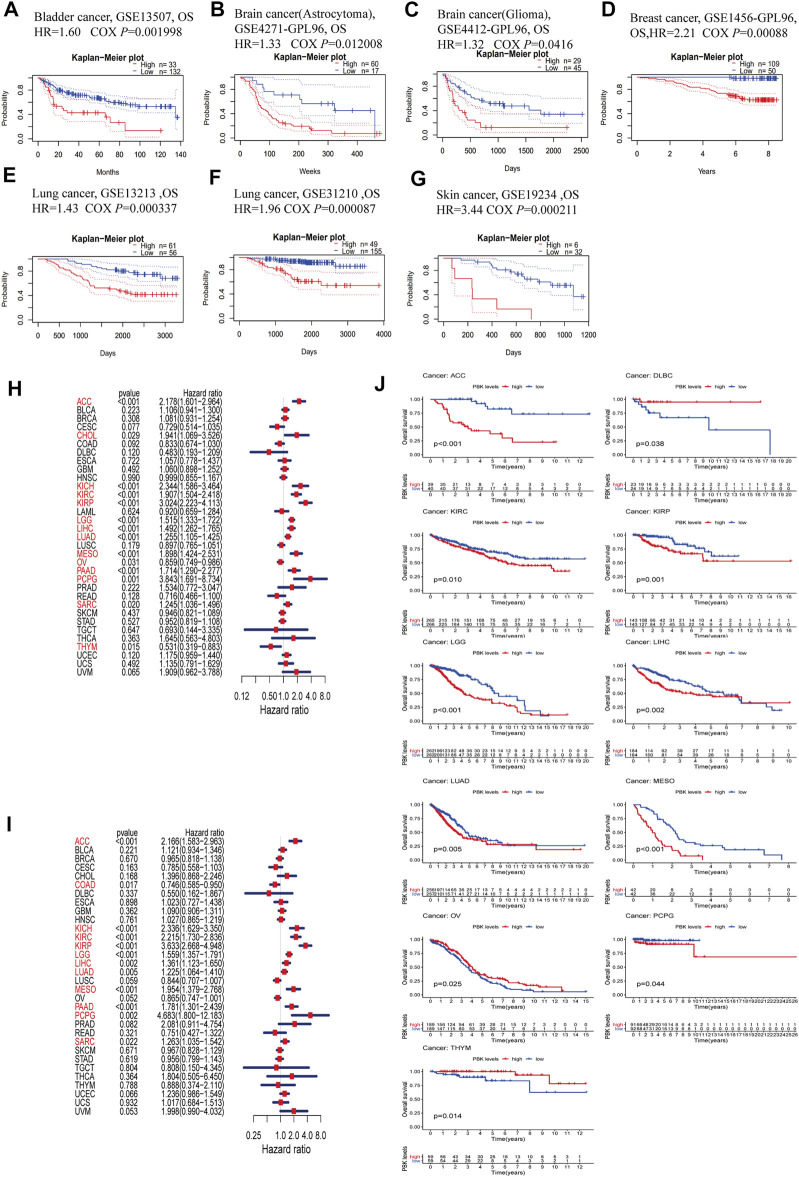
Survival analysis comparing high and low expression of PBK in different types of cancer in the Gene Expression Omnibus and TCGA datasets. **(A–G)** Kaplan–Meier survival curves in 8 cancer cohorts (GSE13507, GSE4271-GP96, GSE4412-GP96, GSE1456-GP96, GSE13213, GSE31210, and GSE19234). **(H, I)** Relationship between PBK expression and patient prognosis (OS and DSS) of different cancers in TCGA database. **(J)** Survival curves of OS in 11 cancer types (ACC, DLBC, KIRC, KIRP, LGG, LIHC, LUAD, MESO, PCPG, OV, and THYM) in TCGA.

To evaluate the prognostic value of PBK expression, we used the PrognoScan database to analyze the relationship between PBK and survival outcomes of cancer patients. The results based on 7 cohorts including GSE13507 (BLCA, HR = 1.60, *p* = 0.001998) (Kim et al., 2010; Lee et al., 2010), GSE4271-GP96 (GBM, HR = 1.33, *p* = 0.012008) (Phillips et al., 2006; Costa et al., 2010; Lin et al., 2011), GSE4412-GP96 (GBM, HR = 1.32, *p* = 0.0416) (Freije et al., 2004), GSE1456-GP96 (BRCA, HR = 2.21, *p* = 0.00088) (Pawitan et al., 2005; Hall et al., 2006), GSE 13213 (LUAD, HR = 1.43,*p* = 0.000337) (Tomida et al., 2009), GSE31210 (LUAD, HR = 1.96, *p* = 0.000087) (Okayama et al., 2012; Yamauchi et al., 2012), and GSE19234 (SKCM, HR = 3.44, *p* = 0.000211) (Bogunovic et al., 2009) showed that high PBK expression was significantly associated with a worse prognosis (Figures 3A–G).

In order to further assess the prognostic value of PBK in cancer, we performed univariate Cox regression and Kaplan–Meier survival analyses using TCGA pan-cancer clinical data from the UCSC database. The results of the univariate Cox regression analysis showed that PBK expression level was associated with clinical outcome in ACC (HR = 2.178, *p* < 0.001), CHOL (HR = 1.941, *p* = 0.029), KICH (HR = 2.344, *p* < 0.001), KIRC (HR = 1.907, *p* < 0.001), KIRP (HR = 3.024, *p* < 0.001), LGG (HR = 1.515, *p* < 0.001), LIHC (HR = 1.492, *p* < 0.001), LUAD (HR = 1.255, *p* < 0.001), mesothelioma (MESO; HR = 1.898, *p* < 0.001), pancreatic adenocarcinoma (PAAD; HR = 1.714, *p* < 0.001), pheochromocytoma and paraganglioma (PCPG; HR = 3.843, *p* = 0.001), and sarcoma (SARC; HR = 1.245) and was a risk factor for OS in PCPG (*p* = 0.02), but a protective factor in OV (HR = 0.859, *p* = 0.031) and THYM (HR = 0.531, *p* = 0.015) (Figure 3H). To avoid deviations caused by non-cancer events, we performed a univariate Cox regression analysis for DSS. PBK was a risk factor for DSS in patients with ACC (HR = 2.166, *p* < 0.001), KICH (HR = 2.336, *p* < 0.001), KIRC (HR = 2.215, *p* < 0.001), KIRP (HR = 3.633, *p* < 0.001), LGG (HR = 1.559, *p* < 0.001), LIHC (HR = 1.361, *p* = 0.002), LUAD (HR = 1.225, *p* = 0.005), MESO (HR = 1.954, *p* < 0.001), PAAD (HR = 1.781, *p* < 0.001), PCPG (HR = 4.683, *p* = 0.002), and SARC (HR = 1.263, *p* = 0.022) but was a protective factor in COAD (HR = 0.746, *p* = 0.017) (Figure 3I).

The Kaplan–Meier survival analysis revealed that high PBK expression predicted worse OS in ACC, KIRC, KIRP, LGG, LIHC, LUAD, MESO, and PCPG but better OS in DLBC, OV and THYM (Figure 3J).

### TFs and miRNAs Regulating PBK

**FIGURE 4:**
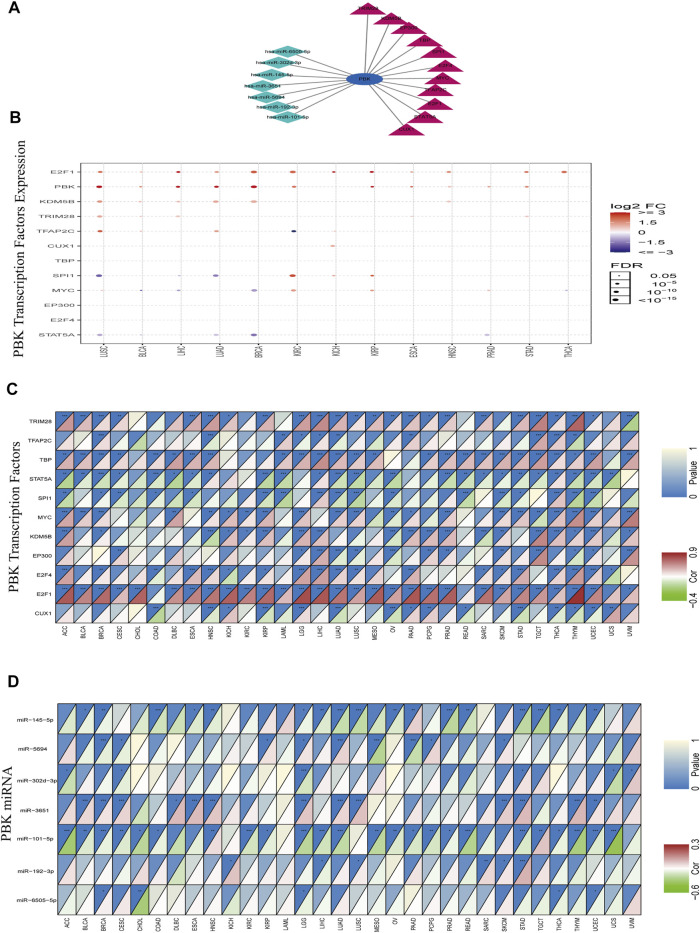
TF and miRNA regulatory networks. **(A)** TF and miRNA network. **(B)** Expression of TFs in 14 cancers. Red and blue dots indicate increased and decreased expression, respectively, in cancers vs normal tissues. **(C, D)** Heatmap of TFs **(C)** and miRNAs **(D)** altered in 32 cancers. Red means TFs or miRNAs is positively correlated with PBK expression, green means TFs or miRNAs is negatively correlated with PBK expression.

Given the importance of PBK in cancer, we explored TFs and miRNAs that regulate PBK expression. We identified 153 transcriptional regulators of PBK in 4 databases including ChEA, NCODE, JASPAR, and TRANSFAC. After screening for those that were represented at least twice in these databases, 11 TFs were ultimately obtained (Figure 4A). We analyzed the expression of these TFs in different cancers using the GSCALite platform. The 11 TFs were highly expressed in most cancers, with E2F1 showing the most significant upregulation (Figure 4B). The heat map indicated that the 11 TFs in 32 cancers were associated with altered PBK expression (Figure 4C): E2F1 in THYM (cor = 0.916), tripartite motif-containing 28 (TRIM28) in THYM (cor = 0.69), MYC in uveal melanoma (UVM) (cor = 0.577), and TATA-binding protein (TBP) in LIHC (cor = 0.497) were positively correlated with PBK level whereas signal transducer and activator of transcription 5A (STAT5A) and Spi-1 proto-oncogene (SPI1) showed negative correlations. E2F1 regulates the cell cycle, DNA repair, apoptosis, and cell proliferation (Fouad et al., 2020). TRIM28 is involved in the regulation of target gene transcription, response to DNA damage, downregulation of p53 activity, stimulation of epithelial–mesenchymal transition (EMT), autophagy induction, and regulation of reverse transcription-mediated transposition (Czerwińska et al., 2017). MYC promotes tumorigenesis by regulating cell proliferation, cell cycle, and genomic instability (Casey et al., 2017). Thus, PBK may contribute to tumorigenesis through interaction with these factors.

MiRNAs control gene expression at the post-transcriptional level and thereby regulate cell proliferation, apoptosis, and invasion in cancer (Li et al., 2018; Zeng et al., 2021). miRNAs targeting PBK were predicted with TargetScan and the miRDB database; a total of 45 miRNAs were obtained in the intersection of the 2 datasets. Correlations between the miRNAs and PBK were calculated using the ENCORI database (*p* < 0.05). Of the 7 miRNAs with significant correlations (hsa-miR-6505-5p, hsa-miR-192-3p, hsa-miR-101-5p, hsa-miR-3651, hsa-miR-302d-3p, hsa-miR-5694, and hsa-miR-145-5p) (Figure 4A), hsa-miR-101-5p in UCS (cor = −0.61), hsa-miR-145-5p in testicular germ cell tumor (TGCT) (cor = −0.357), and hsa-miR-5694 in MESO (cor = −0.368) showed the strongest inhibitory effect on PBK expression (Figure 4D). miR-101-5p is known to inhibit the progression of KIRC, BRCA, non-SCLC (NSCLC), and CESC (Chen Q. et al., 2019; Shen et al., 2019; Toda et al., 2020; Yamada et al., 2020), while miR-145-5p has this effect in GBM and PRAD (Ozen et al., 2015; Kurogi et al., 2018). Our results indicate that these miRNAs negatively regulate PBK expression, thereby affecting its function in tumorigenesis.

### Changes in the Methylation State of PBK Influence Patient Prognosis

**FIGURE 5:**
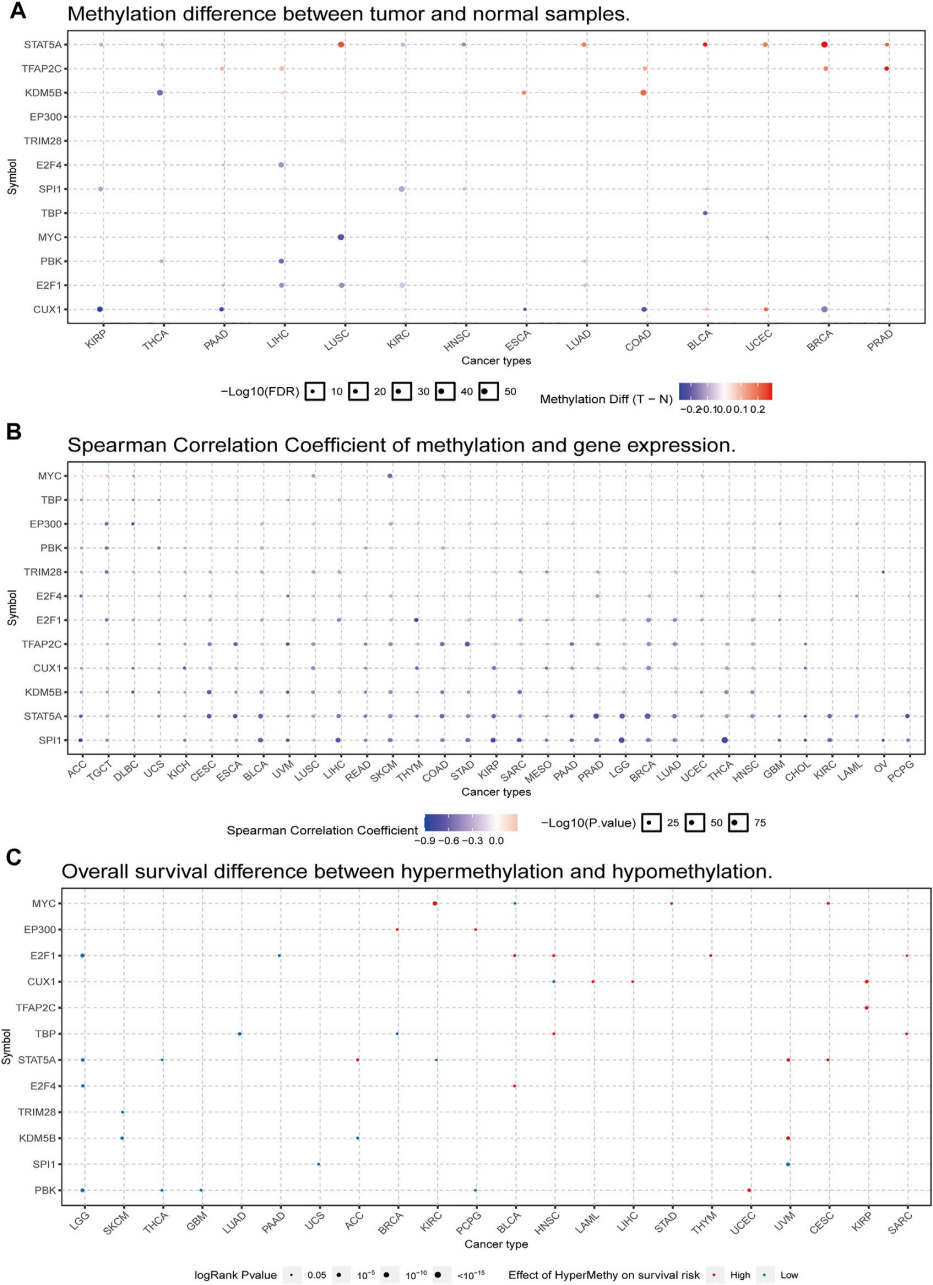
PBK gene methylation in TCGA cancer tissues. **(A)** Bubble map of the differential methylation of PBK and TFs between tumors and corresponding normal tissues in 14 cancer types. Red and blue dots indicate increased and decreased methylation, respectively, in cancers vs normal tissues. **(B)** Bubble map of the association between methylation and expression of PBK and TFs across different cancer types. Red dots represent increased methylation and expression levels, and blue dots represent increased methylation and decreased expression level. **(C)** Bubble map showing the association between OS and methylation of PBK and TFs in different cancer types. Red and blue dots indicate high and low risk, respectively. The size of the dot reflects statistical significance, with a larger size indicating greater significance.

Alterations in DNA methylation state are related to gene expression changes in cancer (Masser et al., 2018). We used the GSCALite platform to analyze the methylation profiles of *PBK* and related TFs in various TCGA cancers. We first compared the methylation status of tumor and normal tissues in 14 cancer types and found that PBK methylation was decreased in THCA, LIHC, and LUAD (Figure 5A). We also examined the correlation between PBK methylation and expression in 32 cancer types and found that the expression levels of PBK and its TFs were negatively correlated with *PBK* methylation, with positive correlations observed only in a few cases (Figure 5B). Finally, we examined the association between PBK methylation and OS in 22 cancer types and found that in LGG, THCA, GBM, and PCPG, a higher methylation level was associated with a lower risk of death, whereas a positive association was observed between PBK methylation and mortality in UCEC (Figure 5C).

### High PBK Expression Is Correlated With Immune Cell Infiltration in Cancers

**FIGURE 6:**
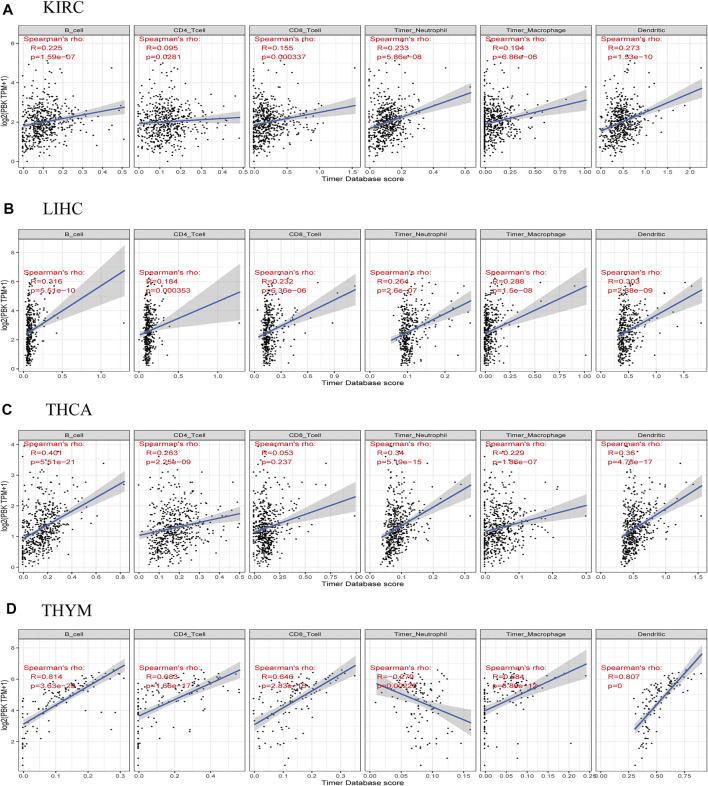
Correlations between PBK expression and immune cell infiltration in cancers. **(A)** KIRC. **(B)** LIHC. **(C)** THCA. **(D)** THYM.

To determine whether PBK is involved in immune cell invasion into the tumor microenvironment, we analyzed the correlation between PBK expression and the infiltration of six immune cell types in 32 cancers in the TIMR database. PBK expression level was positively correlated with the degree of immune cell infiltration in KIRC, LIHC, THCA, and THYM (Figures 6A–D). The correlations between PBK expression and immune cell infiltration in other cancers are shown in Supplementary Figure 1. Our results suggest that in some cancers such as KIRC and LIHC, PBK overexpression can affect patient prognosis by increasing immune cell infiltration.

### Relationship Between Immunotherapy, Immune Checkpoints, and PBK

**FIGURE 7:**
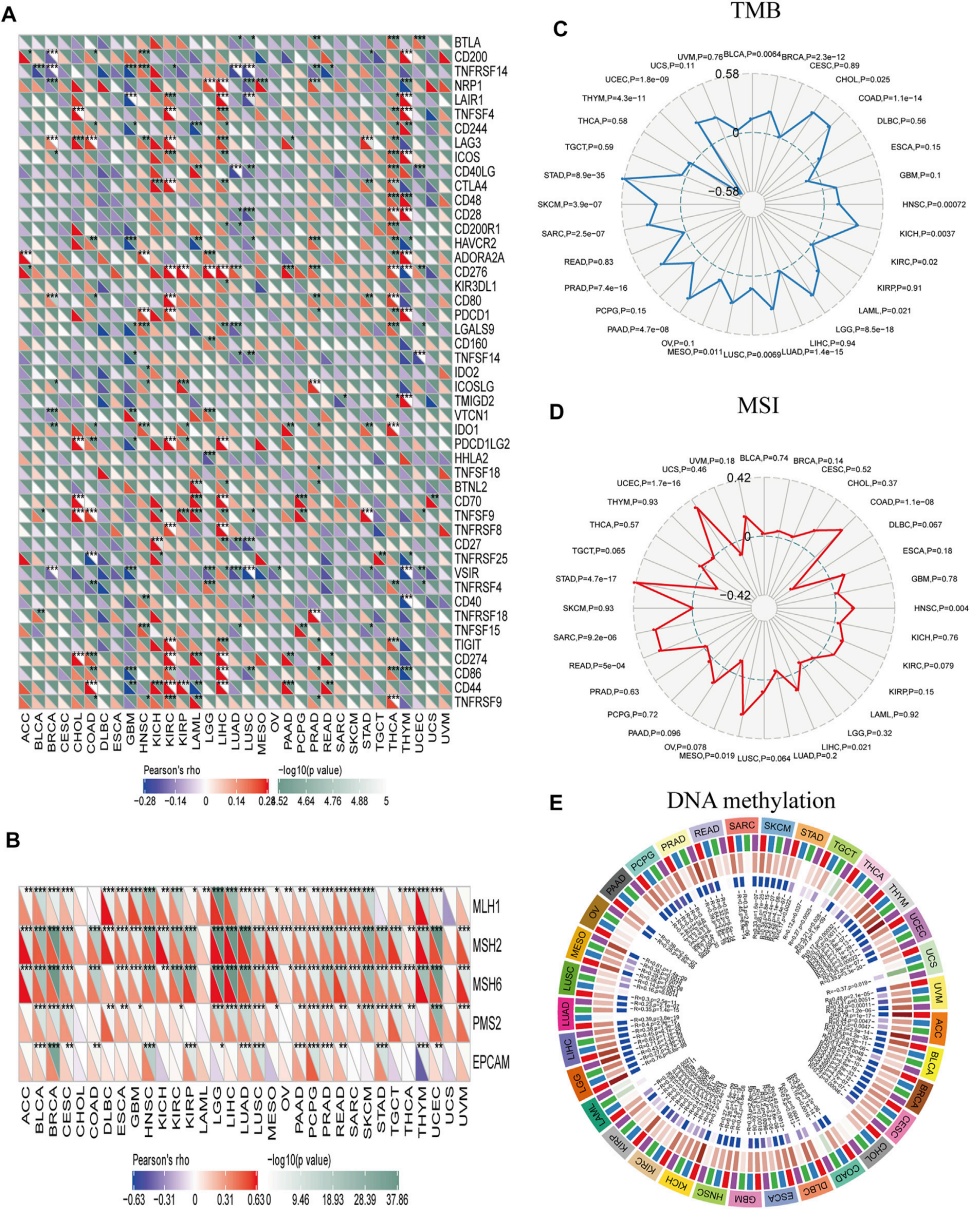
Correlations between PBK and immune checkpoints and other variables of interest. **(A–E)** Correlations between PBK expression and confirmed immune checkpoints **(A)**, genes involved in MMR **(B)**, TMB **(C)**, MSI **(D)**, and DNA methylation **(E)** in multiple cancers. *p < 0.05, **p < 0.01, ***p < 0.001.

Immune checkpoint molecules maintain immune system activation within a normal range; abnormal expression of these molecules is closely related to the occurrence and development of some tumors. Immune checkpoint inhibitors (ICIs) are a treatment option for certain malignancies. Here we analyzed the correlation between PBK expression and immune checkpoint molecules in different cancers. In CHOL, HNSC, KICH, KIRC, LIHC, and THCA, PBK expression was positively correlated with the expression of cluster of differentiation 86 (CD86), T cell immunoreceptor with Ig and ITIM domains (TIGIT), indoleamine 2,3-dioxygenase 1 (IDO1), cytotoxic T-lymphocyte–associated protein 4 (CTLA4), inducible T cell costimulator (ICOS), lymphocyte-activating 3 (LAG3), and tumor necrosis factor superfamily member 4 (TNFSF4). On the contrary, in GBM, LUSC, and THCA, PBK expression was negatively correlated with the expression of immune checkpoint molecules such as CD86, V-set immunoregulatory receptor (VSIR), hepatitis A virus cellular receptor 2 (HAVCR2), and neuropilin 1 (NRP1) (Figure 7A). These results indicate that PBK plays an important role in tumor immune regulation and may thus impact patient survival. Although PBK expression was significant in urothelial cancer, there was no significant difference in expression level between immunotherapy responders and non-responders (Supplementary Table 1).

Defects in MMR and subsequent MSI can lead to the accumulation of mutations that increase TMB, which in turn stimulates the host’s antitumor immune response (Zhao et al., 2019). We examined the correlation between PBK expression and MMR signatures and found that PBK level was positively correlated with MutL homolog 1 (MLH1), MSH2, MSH6, PMS1 homolog 2 (PMS2), and epithelial cell adhesion molecule (EPCAM) levels in BLCA, BRCA, CESC, LGG, LUAD, LUSC, PAAD, PCPG, PRAD, READ, and UCEC. It was also negatively correlated with EPCAM and positively correlated with MLH1, MSH2, and MSH6 in THYM (Figure 7B). PBK score was positively correlated with TMB in BLCA, BRCA, CHOL, COAD, HNSC, KICH, KIRC, UCEC, LAML, LGG, LUAD, LUSC, MESO, PAAD, PRAD, SARC, SKCM, and STAD; but negatively correlated with TMB in THYM (*p* < 0.05; Figure 7C). Finally, PBK expression was positively correlated with MSI in COAD, HNSC, LIHC, MESO, READ, SARC, STAD, and UCEC (*p* < 0.05; Figure 7D) and with the expression of DNA methyltransferases (DNMT1, DNMT2, DNMT3A, and DNMT3B) in ACC, BLCA, BRCA, CESC, ESCA, KICH, KIRC, KIRP, LGG, LIHC, MESO, PCPG, SKCM, THYM, UCEC, and UVM (*p* < 0.05; Figure 7E).

### Enrichment of PBK-Binding Protein Networks

**FIGURE 8:**
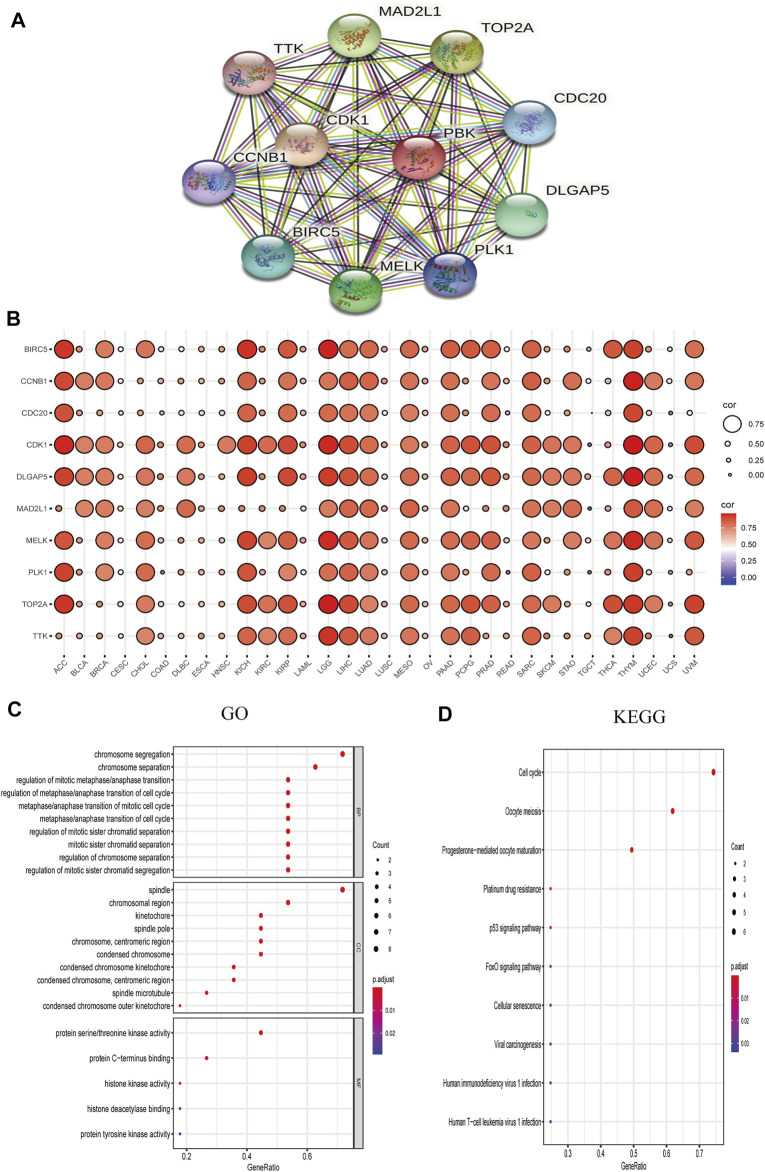
PBK network. **(A)** PBK correlation network. **(B)** Correlation heatmap of 10 genes interacting with PBK in 32 cancers. **(C, D)** GO (biological process [BP], cellular component [CC], and molecular function [MF]) **(C)** and KEGG pathway enrichment **(D)** analyses of the PBK correlation network.

We explored the functional enrichment of PBK in cancer by mapping the protein–protein interaction network of PBK with the STRING database. Ten genes including baculoviral IAP repeat-containing 5 (BIRC5), cyclin B1 (CCNB1), cell division cycle protein 20 (CDC20), cyclin-dependent kinase 1 (CDK1), DLG-associated protein 5 (DLGAP5), mitotic arrest deficient 2-like 1 (MAD2L1), maternal embryonic leucine zipper kinase (MELK), polo-like kinase 1 (PLK1), DNA topoisomerase II alpha (TOP2A), TTK protein kinase (TTK), and PDZ-binding kinase (PBK) formed an interaction network with PBK (Figure 8A). We analyzed the association between these 10 genes and PBK in 32 TCGA cancers and found that the expression of all of the genes in all of the cancers was positively correlated with PBK expression (Figure 8B). The results of the GO functional enrichment analysis showed that PBK is involved in the regulation of cell cycle, serine/threonine kinase activity, and cell division (Figure 8C). The KEGG pathway analysis results revealed enrichment in “cell cycle,” “p53 signal pathway,” and “cell senescence” (Figure 8D). Thus, PBK has potential functions in cell cycle regulation.

### Drug and Pathway Activity Analysis

**FIGURE 9:**
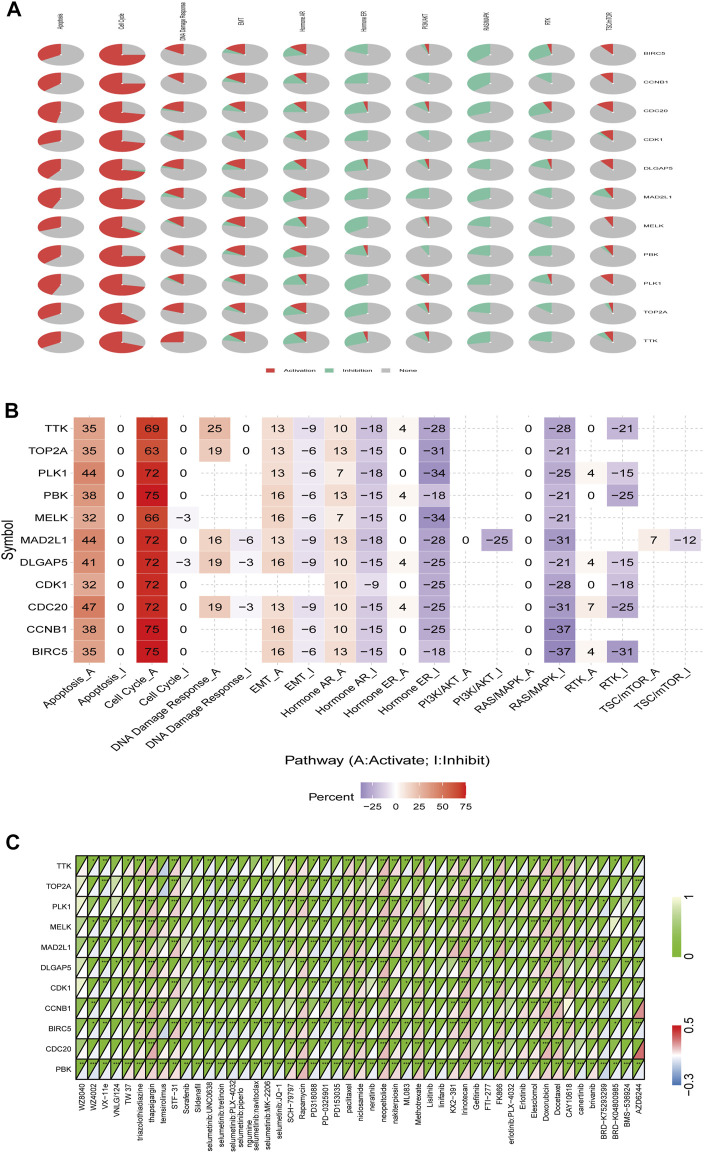
Role of PBK in known cancer-related pathways (GSCALite) and analysis of drugs potentially targeting PBK based on PharmacoDB data. **(A, B)** Pie chart **(A)** and heatmap **(B)** of pathway activity. **(C)** Heatmap of drugs correlated with 10 genes (BIRC5, CCNB1, CDC20, CDK1, DLGAP5, MAD2L1, MELK, PLK1, TOP2A, and TTK) and PBK.

We examined the relationship between the expression of PBK and its interaction partners and the activation or inhibition of cancer-related signaling pathways using the GSCALite platform. Consistent with results of the functional enrichment analysis, BIRC5, CCNB1, CDC20, CDK1, DLGAP5, MAD2L1, MELK, PLK1, TOP2A, TTK, and PBK were related to the activation of apoptosis, cell cycle, and DNA damage response; and BIRC5, CCNB1, CDC20, CDK1, DLGAP5, MAD2L1, MELK, PLK1, TOP2A, TTK, and PBK were related to the inhibition of hormone receptors and RAS/MAPK pathways. Additionally, PBK was related to the induction of EMT as well as activation of TSC/mammalian target of rapamycin (mTOR) and inhibition of PI3K/AKT and receptor tyrosine kinase (RTK) signaling pathways (Figures 9A,B).

We searched the PharmacoDB database for drugs related to PBK with a cutoff of *p* < 0.01. Drugs without targets molecules or pathways were excluded based on standard coefficients >0.1 and <−0.1. PBK was found to be resistant to 31 drugs and sensitive to 20 including those targeting the cell cycle such as paclitaxel, docetaxel, doxorubicin, and irinotecan; and apoptosis regulators such as brivanib, TW37, and elesclomol (Figure 9C).

The mTOR signaling pathway regulates cell metabolism, growth, proliferation, survival, and autophagy in many cancers; mTOR inhibitors suppress inflammation, proliferation, autophagy, and apoptosis and are used in cancer treatment (Chen and Zhou, 2020). PBK was found to be sensitive to rapamycin and temsirolimus, which target the mTOR signaling pathway.

PBK overexpression was associated with resistance to linifanib, lisitinib, neratinib, erlotinib, BRD-K04800985, VX-11e, sildenafil, PD-0325901, erlotinib, gefitinib, AZD6244, VNLG/124, WZ8040, PD318088, BMS-536924, FTI-277, canertinib, PD153035, sorafenib, ML083, WZ4002, and BRD-K75293299.

## Discussion

PBK has been described in some human cancers and its overexpression is associated with tumor invasion, proliferation, and metastasis in OV, lung cancer, COAD, and PRAD (Shih et al., 2012; Chen et al., 2015; Ikeda et al., 2016; Zykova et al., 2017). However, the function of PBK in many other tumor types remains unclear. In this study, we provided evidence for PBK as a pan-cancer biomarker based on gene expression and survival data and analyses of immune cell infiltration, TFs, miRNAs, pharmacogenomics, and related intracellular signaling pathways.

The TCGA and GTEx data showed that PBK was highly expressed in ACC, BLCA, BRCA, CESC, CHOL, COAD, ESCA, GBM, HNSC, KICH, KIRC, KIRP, LGG, LIHC, LUAD, LUSC, OV, RAAD, PRAD, SKCM, STAD, THCA, UCEC, and UCS and downregulated in LAML compared to normal tissue. The Kaplan–Meier survival analysis revealed that high expression of PBK was a risk factor in ACC, DLBC, KIRC, KIRP, LGG, LIHC, LUAD, MESO, and PCPG and a protective factor in OV and THYM. Additionally, we found that PBK expression was positively correlated with cancer stage in ACC, KICH, KIRC, KIRP, and LUAD. PBK expression level was shown to be significantly associated with the progression of ACC and NSCLC (Lei et al., 2013; Kar et al., 2019). Thus, PBK is a prognostic biomarker in many cancers, especially in ACC and LUAD as well as in KIRC and KIRP, which has not been previously reported.

The expression of TFs related to PBK showed the highest positive correlations with UVM and THYM, while PBK-related miRNAs had the strongest inhibitory effects in UCS, TGCT, and MESO. These factors are involved in the regulation of the cell cycle, apoptosis, and cell proliferation and are linked to genome instability. For example, MYC is an oncogene in many cancers and plays an important role in regulating metabolic pathways, especially glutamine and glutamine breakdown (Tambay et al., 2021). TFs regulating PBK expression may thus influence its function in tumorigenesis.

PBK methylation was decreased in THCA, LIHC, and LUAD, which was negatively correlated with the expression level of PBK. Additionally, PBK methylation level was correlated with OS in LGG, THCA, GBM, PCPG, and UCEC cancer. The correlation was especially strong for THCA; therefore, while PBK expression cannot be used as a prognostic marker for THCA, we show here for the first time that a change in its methylation status may predict survival outcome.

There have been few studies on the function of PBK in the tumor immune microenvironment. We observed a negative correlation between PBK expression and the infiltration of immune cells including B cells, CD4^+^ T cells, CD8^+^ T cells, and macrophages in most tumors, but this association was especially strong in KIRC, LIHC, THCA, and THYM. Based on this observation combined with the survival data, we speculate that PBK affects the prognosis of cancers—particularly KIRC and LIHC—by enhancing immune cell infiltration.

ICIs are a type of immunotherapy with demonstrated efficacy and safety in multiple cancers (He and Xu, 2020). Patients with high TMB or MSI are more likely to experience long-term survival benefits from immunotherapy (Samstein et al., 2019; Zhu et al., 2021). To date there have been no studies on the relationship between TMB and MSI and PBK expression in cancers. Our results showed that PBK expression in various types of tumor was related to TMB and MSI, although additional studies are needed in order to determine the significance of this observation in terms of response to immunotherapy.

Functional enrichment analysis showed that PBK and related genes were functionally enriched in cell cycle regulation. Previous studies have shown that PBK binds to the CDK1/cyclin B1 complex on the mitotic spindle and induces its phosphorylation, leading to cell proliferation and cell cycle progression (Côté et al., 2002; Park et al., 2010). Dysregulation of the cell cycle results in abnormal cell proliferation, inhibition of apoptosis, and increased invasion and metastasis (Zaretsky et al., 2016). Thus, PBK may play an oncogenic role by interfering with cell cycle regulation. This finding can guide the development of targeted drugs for cancer treatment.

BIRC5, CCNB1, CDC20, CDK1, DLGAP5, MAD2L1, MELK, PLK1, TOP2A, TTK, and PBK formed an interaction network that was associated with activation of cell apoptosis and the DNA damage response and inhibition of RAS/MAPK signaling. PBK was also related to the induction of EMT, activation of TSC/mTOR signaling, and inhibition of the PI3K/AKT and RTK pathways. Thus, PBK and PBK-related genes promote tumorigenesis through a variety of mechanisms.

The low solubility and toxicity of the PBK inhibitors HI-TOPK-032 and OTS964TOPK limit their clinical application (Matsuo et al., 2014; Ikeda et al., 2016). As such, new PBK inhibitors for cancer treatment are needed. In this study, we screened 31 drugs that potentially target PBK and identified several candidates including linifanib, which is used in the treatment of LIHC, COAD, STAD, NSCLC, and HNSC (Hsu et al., 2014; Chen et al., 2016; Horinouchi, 2016; Chan et al., 2017; Han et al., 2021); neratinib, which is used in BRCA (Saura et al., 2021); erlotinib and gefitinib, which are used in NSCLC (Yang et al., 2017); and BMS-536924, which is used in GBM and ESCA (Adachi et al., 2014; Zhou, 2015). The anticancer effect of these drugs may be related to PBK inhibition.

## Conclusion

We carried out a comprehensive evaluation of PBK to investigate its potential roles in promoting cancer and as a prognostic indicator through bioinformatics analyses. We identified 4 TFs and 3 miRNAs that were related to PBK upregulation and downregulation, respectively, in cancer. We also found that PBK expression is related to cancer immunity: in some cancers, PBK may affect tumor progression by increasing immune cell infiltration into the tumor microenvironment, making it a potential target for cancer immunotherapy. Functional enrichment analysis showed that PBK and its related genes regulate tumor-related signaling pathways. Finally, we found that PBK is associated with sensitivity and resistance to various anticancer drugs and is related to drug-targeted genes in tumor cells. These findings indicate that evaluating PBK expression levels may have diagnostic utility in cancer and can guide treatment decisions, although experimental studies are needed to confirm this possibility.

## Data Availability

The datasets presented in this study can be found in online repositories. The names of the repository/repositories and accession number(s) can be found in the article/Supplementary Material.

## Glossary

ACC: adrenocortical carcinoma
BLCA: bladder urothelial carcinoma
BRCA: breast invasive carcinoma;
CESC: cervical squamous cell carcinoma and endocervical adenocarcinoma
CHOL: cholangiocarcinoma
COAD: colon adenocarcinoma
DLBC: lymphoid neoplasm diffuse large B-cell lymphoma
ESCA: esophageal carcinoma
GBM: glioblastoma multiforme
HNSC: head and neck squamous cell carcinoma
KICH: kidney chromophobe
KIRC: kidney renal clear cell carcinoma
KIRP: kidney renal papillary cell carcinoma
LAML: acute myeloid leukemia
LGG: brain lower grade glioma
LIHC: liver hepatocellular carcinoma
LUAD: lung adenocarcinoma
LUSC: lung squamous cell carcinoma
MESO: mesothelioma
NSCLC: non-small cell lung cancer
OV: ovarian serous cystadenocarcinoma
PAAD: pancreatic adenocarcinoma
PCPG: pheochromocytoma and paraganglioma
PRAD: prostate adenocarcinoma
READ: rectum adenocarcinoma
SARC: sarcoma
SKCM: skin cutaneous melanoma
STAD: stomach adenocarcinoma
TGCT: testicular germ cell tumor
THCA: thyroid carcinoma
THYM: thymoma
UCEC: uterine corpus endometrial carcinoma
UCS: uterine carcinosarcoma
UVM: uveal melanoma.
