# Supplementary figures and images for: Omics- and Pharmacogenomic Evidence for the Prognostic, Regulatory, and Immune-Related Roles of PBK in a Pan-Cancer Cohort
Source: Front Mol Biosci. 2021 Nov 11;8:785370. doi: 10.3389/fmolb.2021.785370 (PMC8632063; doi:10.3389/fmolb.2021.785370)

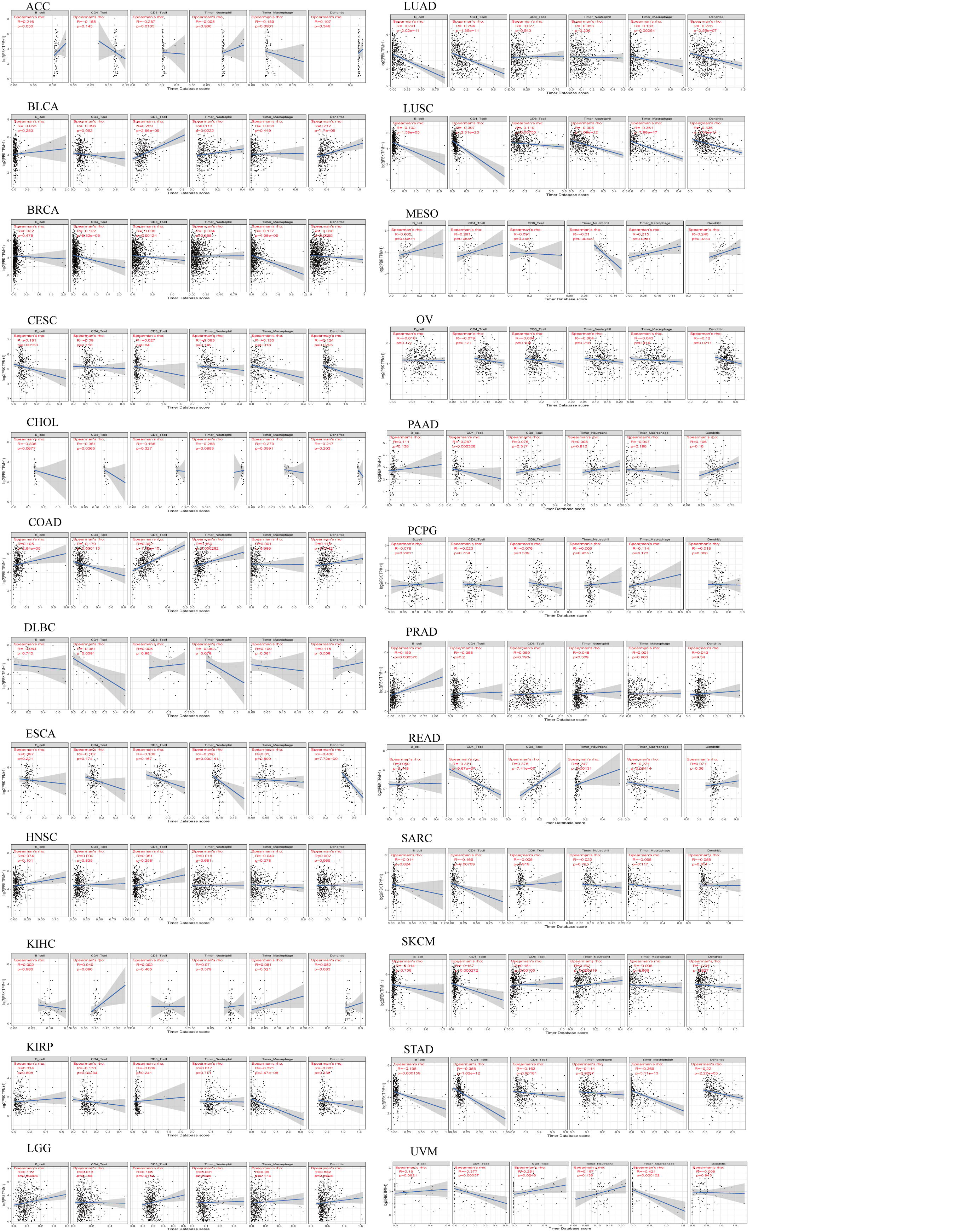

Supplement: Supplementary file 1 [file Image1.TIF]
